# Sacrificial-layer free transfer of mammalian cells using near infrared femtosecond laser pulses

**DOI:** 10.1371/journal.pone.0195479

**Published:** 2018-05-02

**Authors:** Jun Zhang, Bastian Hartmann, Julian Siegel, Gabriele Marchi, Hauke Clausen-Schaumann, Stefanie Sudhop, Heinz P. Huber

**Affiliations:** 1 Lasercenter, Munich University of Applied Sciences, Lothstrasse, Munich, Germany; 2 Center for Applied Tissue Engineering and Regenerative Medicine CANTER, Munich University of Applied Sciences, Lothstrasse, Munich, Germany; 3 Center for NanoScience, University of Munich, Munich, Germany; 4 Experimental Trauma Surgery, Department of Trauma Surgery, Franz-Josef-Strauss-Allee, Regensburg, Germany; 5 Photonics Laboratory, Munich University of Applied Sciences, Lothstrasse, Munich, Germany; Louisiana State University Health Sciences Center, UNITED STATES

## Abstract

Laser-induced cell transfer has been developed in recent years for the flexible and gentle printing of cells. Because of the high transfer rates and the superior cell survival rates, this technique has great potential for tissue engineering applications. However, the fact that material from an inorganic sacrificial layer, which is required for laser energy absorption, is usually transferred to the printed target structure, constitutes a major drawback of laser based cell printing. Therefore alternative approaches using deep UV laser sources and protein based acceptor films for energy absorption, have been introduced. Nevertheless, deep UV radiation can introduce DNA double strand breaks, thereby imposing the risk of carcinogenesis. Here we present a method for the laser-induced transfer of hydrogels and mammalian cells, which neither requires any sacrificial material for energy absorption, nor the use of UV lasers. Instead, we focus a near infrared femtosecond (fs) laser pulse (*λ* = 1030 nm, 450 fs) directly underneath a thin cell layer, suspended on top of a hydrogel reservoir, to induce a rapidly expanding cavitation bubble in the gel, which generates a jet of material, transferring cells and hydrogel from the gel/cell reservoir to an acceptor stage. By controlling laser pulse energy, well-defined cell-laden droplets can be transferred with high spatial resolution. The transferred human (SCP1) and murine (B16F1) cells show high survival rates, and good cell viability. Time laps microscopy reveals unaffected cell behavior including normal cell proliferation.

## Introduction

Laser-induced transfer–also referred to as laser printing–is a promising direct write technology that can rapidly and flexibly print materials with high spatial resolution [1]. It was originally developed to transfer inorganic materials from a thin donor film to an acceptor surface by means of laser pulses focused on the donor film through a transparent support [2]. In recent years, laser-induced transfer has also been applied to biological material as an alternative bio-printing technology. In this context the term laser assisted bioprinting (LAB) was introduced. It can overcome some of the drawbacks of the more conventional ink-jet printing, pipetting, and micro-extrusion based technologies, such as clogging of printing nozzles, or high shear forces. Because printer parts do not come into direct contact with printing material, cross-contamination of different materials can easily be avoided. In addition, owing to the high repetition rates of pulsed laser sources, laser printing has the potential for high transfer rates and fast processing times.

In the past, biomolecules [3], like proteins [4,5] or DNA [5–7], as well as mammalian cells [8–14] have been successfully transferred through laser printing with almost no loss of bioactivity. In a typical setup for laser-induced cell transfer, a transparent substrate is coated with a light absorbing layer such as gold, titanium [8,9,11,13] or a light absorbing polymer [15–17]. The cell-containing hydrogel is deposited onto the absorbing layer with a typical thickness of about 100 μm. The absorbing layer is then evaporated by focusing a laser pulse through the transparent substrate into the absorbing layer, resulting in an evaporation of the absorbing layer and a high gas pressure, which propels the biomaterial towards an acceptor surface. The transferred cells usually display a high survival rate and maintain their ability to proliferate [8,11]. Scaffold-free 3D cell microstructures for cell-cell and cell-substrate interaction studies and tissue engineering applications have been successfully fabricated in this manner [8,9,11].

One drawback of laser based transfer for bioprinting applications, such as cell printing and tissue engineering is the fact, that material from the energy absorbing layer is transferred along with the printed biomaterial, contaminating the printed constructs, where it can be found in the form of nanometer and larger fragments and particles [5,18]. To avoid contamination of constructs with inorganic material, protein hydrogels, such as Matrigel or collagen hydrogels, have been used as light absorbing layer [17], as used in matrix-assisted pulsed-laser evaporation direct writing (MAPLE DW) [10,19,20]. Nevertheless, these approaches are limited to UV laser irradiation, such as emitted from argon fluoride excimer lasers (193 nm), because they rely on the effective UV absorption of proteins at wavelengths at and below 200 nm [21]. However, at these wavelengths, UV light may cause severe DNA damage, including double strand breaks [17] and photochemical crosslinking, both of which may lead to cell death or carcinogenesis [22].

In the present study, we therefore present an alternative approach, which avoids both, the use of non-biological, inorganic absorption layers and of UV-lasers sources, which are prone to induce DNA damage, thereby imposing the risk of carcinogenesis. Focused femtosecond laser pulses provide the high photon densities, which lead to a spatially confined optical breakdown with very efficient energy absorption without the need for light absorbing layers [23–28]. In addition, we use the near infrared window, where the interaction of radiation with biological material is minimal [21,22], thereby avoiding the risk of inducing photochemical DNA damage. In aqueous media, the high pressure plasma generated by the ultrashort laser pulses forms a rapidly expanding cavitation bubble [29]. When the femtosecond laser focus is placed to a focus depth of 50 μm to 100 μm underneath the liquid surface, the cavitation bubble can be used to propel a water or hydrogel jet, which is subsequently ejected from the free liquid surface [30,31]. It has been shown that the fast expansion of the cavitation bubble, effectively converts the laser pulse energy into kinetic energy avoiding heat transfer to the surrounding medium [26,32]. Current applications of this technique which focus on the controllable and reproducible transfer of biomolecules like proteins and DNA showed no loss of bioactivity [33,34], indicating that this approach might also be feasible for the printing of living cells. We have therefore adapted this approach for the transfer of living mammalian cells and tested its feasibility, using human mesenchymal stem cells (hMSC, SCP1 cell line) and murine skin melanoma cells (B16F1 cell line). We obtained high survival rates of transferred cells, and in addition, cells retained their full viability, showing cell-line specific behavior with normal proliferation rates and cell motility.

## Materials and methods

### Cell culture

Mouse skin melanoma B16F1 cells were obtained from ATCC (Wesel, Germany). SCP1 cells are immortalized human mesenchymal stem cells, described and fully characterized in Böker et al. 2008 [35]. Both cell lines were maintained in Dulbeccos modified eagles medium (DMEM, Biochrom, Germany) supplemented with 10% fetal bovine serum (Biochrom, Germany), 1% GlutaMAX (Thermo Fisher Scientific) and 1% Penicillin/Streptomycin (Biochrom, Germany). In routine cell culture, all cells were grown up to 80% confluency and maintained at 37°C in 10% humidified CO_2_ in a T175 cell culture flask. For cell passaging, cells were detached with 0.25% trypsin/0.02% EDTA solution (Biochrom, Germany).

### Preparation of the hydrogel reservoir and acceptor surface

The hydrogel for laser-induced transfer, was produced by dissolving 0.2% alginic acid sodium salt from brown algae (Sigma-Aldrich, Germany) in histopaque-1083, a gradient medium with a density of 1.083 g/ml (Sigma-Aldrich, Germany). For preparation of cell-laden gels, 5×10^6^ SCP1-cells or 20×10^6^ B16F1 cells, respectively, were harvested, the resulting cell pellet was resuspended in 2 ml of the further mentioned hydrogel. This suspension was transferred into a petri dish, which served as reservoir, and incubated at room temperature for 15 minutes, to allow the cells to rise to the hydrogel surface. In the meantime, a second petri dish (TC Dish 60, Standard, Sarstedt, Germany) was prepared as an acceptor surface. The interior was covered with a thin film of gelatin (Sigma-Aldrich, Germany), when using SCP1 cells, or Matrigel (BD Biosciences, Heidelberg) for B16F1 cells to cushion the impact of landing cells, and to maintain a humidified environment to protect the transferred cells from drying out [12]. In addition, the coating with extracellular matrix proteins helped to facilitate cell adhesion to the substrate. The gelatin film was prepared by dissolving gelatin in PBS (10% w/v) at about 50°C. 30 μl of this solution were homogenously dispersed on the bottom of the petri dish and then cooled to 2°C for 15 min, whereby the gelatin formed a film of about 100 μm thickness. For Matrigel-coating, the gel was thawed at 4°C overnight, 30 μl of cold Matrigel were evenly dispersed in the pre-cooled petri dish and then incubated at 37°C for 10 min to obtain a 100 μm layer.

### Laser setup

**Fig 1:**
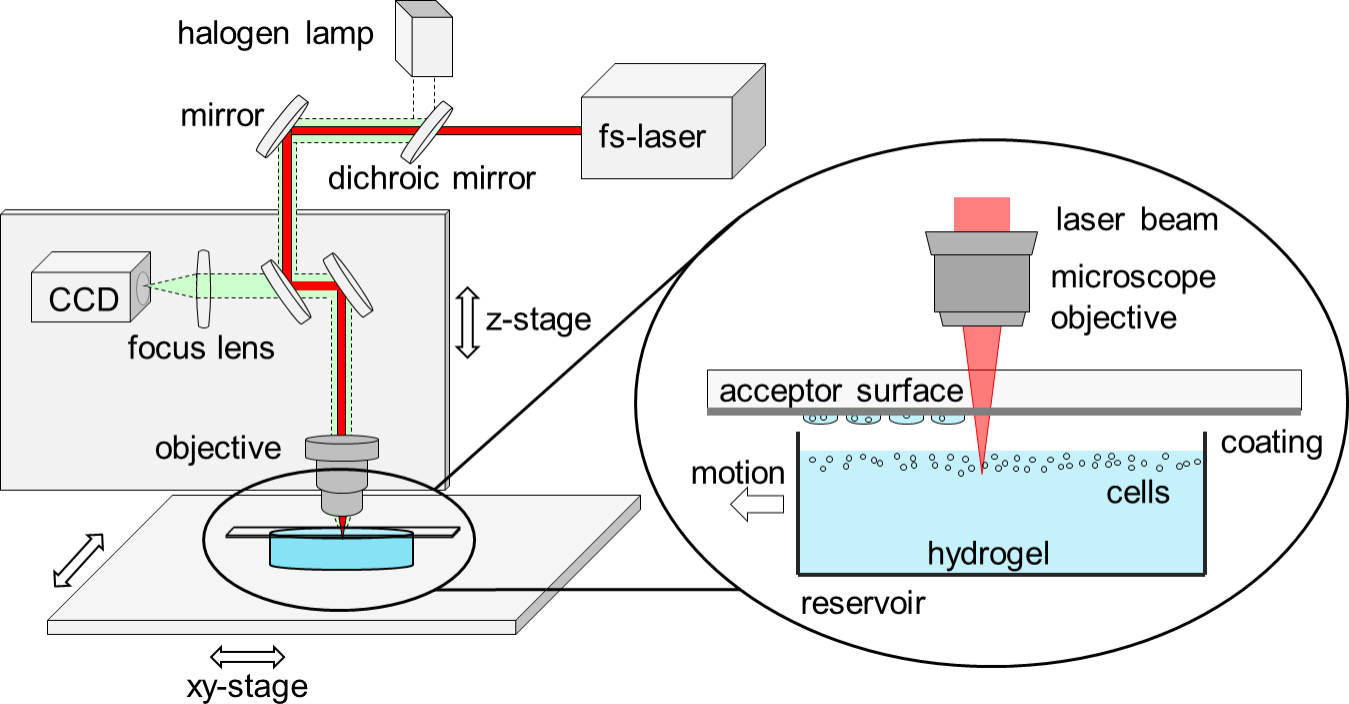
Schematic representation of the cell transfer setup. The fs-laser beam is focused through the transparent acceptor petri dish into the reservoir containing the cell-laden hydrogel. The cells accumulate at the hydrogel surface due to the density of histopaque-1083 used for gel preparation. The focus depth is chosen to be between 50 and 65 μm and is therefore located directly beneath the cells. The highly confined optical breakdown generates a rapidly expanding cavitation bubble, which ejects a cell-laden hydrogel jet towards the acceptor slide.

For the laser-induced cell transfer setup, an industrial Yb:KYW (potassium yttrium tungstate) femtosecond laser (Spectra Physics, Austria) with 1030 nm wavelength, 450 fs pulse duration and 10 μJ maximum pulse energy at laser exit was chosen. It relies on diode-pumped saturable-absorber mode-locking technology for robust performance and provides high output power than commonly scientific ultrafast Ti:sapphire lasers. In addition, the emission wavelength of 1030 nm lies in the near-infrared window of biological tissue. Yb:KYW femtosecond lasers are currently used in high quantities in medicine, life-science and industry. The collimated laser beam was focused through a transparent acceptor petri dish into the hydrogel, using a 32× microscope objective (Leica Wetzlar, Germany) with a numerical aperture of 0.6, a transmittance of 65% at 1030 nm wavelength, and a working distance of 6 mm (cf. Fig 1). In order to control the x-y-position of the transferred hydrogel micro-droplets, both hydrogel reservoir and acceptor petri dish were mounted on an x-y-stage (Laser Systems GmbH, Germany). To avoid undesired wave formation on the hydrogel surface, the motion of the stage was limited to velocities ≤ 1 mm/s. The height of the microscope objective was controlled by a z-stage, which allowed to vary focus depth in the liquid. For all experiments shown, we used focus depths between 50 and 65 μm. The distance between the hydrogel/cell surface and the acceptor petri dish was 500 μm, unless stated otherwise.

By focusing the beam to a molybdenum surface and applying the d^2^-method, which is measuring the diameter of the resulting ablation spots at various pulse energies, the diameter of the laser focus in this set up was determined to be 1.6 μm at 1/e^2^ of maximum intensity [36]. This is leading to a peak intensity of ≈ 10^15^ W/cm^2^ after the microscope objective at full laser pulse energy, which is well above the threshold for optical breakdown in aqueous solution of 10^12^−10^13^ W/cm^2^ [26]. Above this threshold, multi-photon absorption leads to plasma ionization and the generation of a rapidly expanding cavitation bubble, which ejects a hydrogel jet from the surface towards the acceptor petri dish, resting above the hydrogel reservoir [30].

### Analysis of cell viability

To evaluate the survival rate of transferred cells, the gelatin layer on the acceptor slide was supplemented with Propidium Iodide ReadyProbes reagent (PI R37108, Thermo Fischer, Germany). Intact cells reject propidium iodine (PI), in dead cells PI binds to DNA and causes a red fluorescence. For this purpose, 2 drops of PI were added to 1 ml gelatin solution before dispersing on the acceptor petri dish. After laser-induced transfer, the acceptor petri dish containing the cell-laden hydrogel droplets was incubated at 37°C in 10% humidified CO_2_ for 15 min to allow PI staining. Dead cells were visualized by fluorescence microscopy using an inverted optical microscope (Observer Z.1, Carl Zeiss, Göttingen, Germany).The viability of non-transferred cells remaining in the reservoir was also investigated by PI staining: Live and dead cells were counted using a standard hemocytometer chamber in the fluorescence microscope.

### Analysis of cell proliferation after laser-induced transfer

Immediately after transfer, B16F1 cells transferred to Matrigel-coated acceptor petri dishes were placed in an incubation-chamber, providing 37°C and 10% humidified CO_2_ atmosphere (Pecon, Erbach). This chamber was mounted on an inverted optical microscope, and a first image was recorded. After 15 min, 3 ml of DMEM cell culture medium were gently added. From now on, microscopy images were collected in 20 min intervals for about 40 hours. The images were taken using an Orca Flash 4.0 scientific CMOS camera (Hamamatsu, Herrsching, Germany).

## Results and discussion

**Fig 2:**
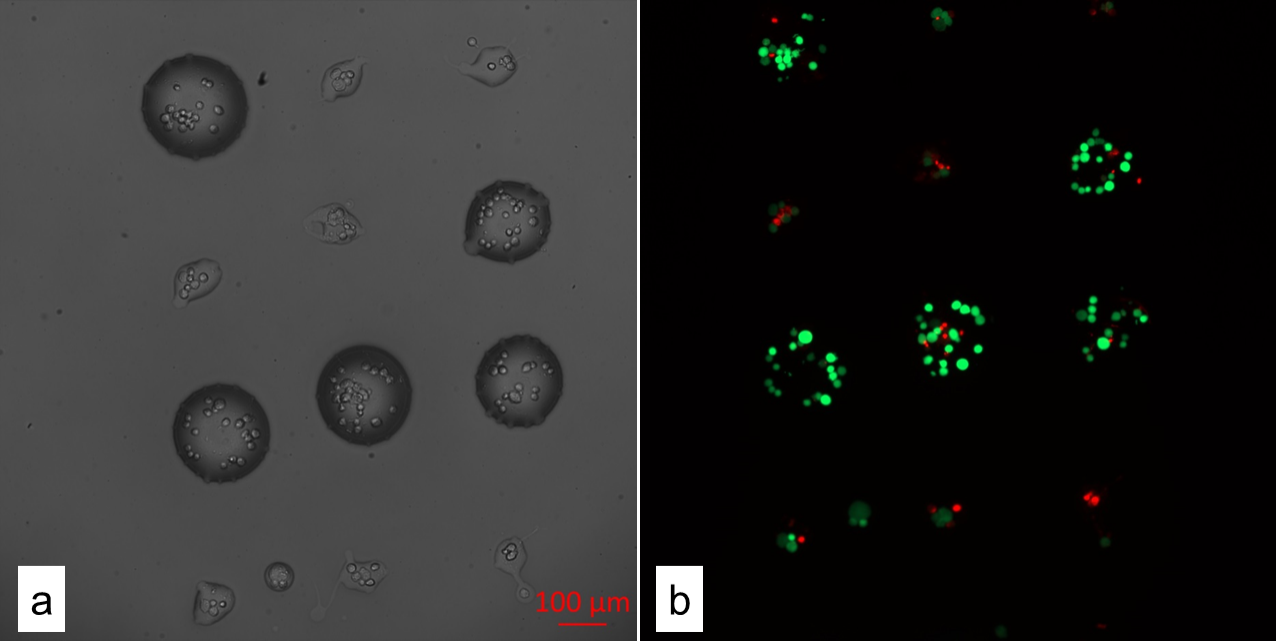
Representative microscope images of cell-laden hydrogel droplets. (a) In the bright field image, the large droplets show a diameter of about 200 μm, while the small droplets size up to a diameter of only 80 μm. (b) The fluorescence image reveals a cell survival of up to 91 ± 2% in the larger droplets (red PI staining indicates dead cells, live cells are displayed in green), in small droplets only 62 ± 14% of cells survive the laser-induced transfer.

Fig 2 shows bright field (a) and fluorescence (b) images of hydrogel micro-droplets (0.2% alginate in histopaque) containing GFP labelled SCP1 cells, after laser-induced cell transfer to a gelatin coated acceptor petri dish. The laser pulse energy after the microscope objective was 5.0 μJ, the focus depth 50 μm and the distance between liquid and acceptor dish 500 μm. The large droplets are circular with an average diameter of about 200 μm, containing 20 ± 5 (mean value and standard deviation) cells each, while the smaller droplets have a more irregular appearance, and an average diameter of about 80 μm, containing only 5.6 ± 2 cells each. In the fluorescence image, live cells appear in green, while dead cells appear in red, because of the PI staining (cf. materials and methods for details). In the large droplets, the cell survival rate is 91 ± 2%, while in the small irregular droplets the survival rate is only 62 ± 14%. For comparison, the survival rate of cells remaining in reservoir is 92 ± 1% (data not shown). The mean values and the standard deviations of the cell number per droplet and their survival rate, respectively, was calculated for n = 73 larger and n = 146 smaller droplets obtained from 5 independent experiments.

High survival rates and good cell viability are key parameters of biofabrication techniques for tissue engineering. In laser-induced cell transfer the most important causes of cell damage are high acceleration forces caused by the expanding cavitation bubble, shear forces, as cells are moving through the hydrogel, and forces caused by the impact of the cell-laden droplets on the acceptor [14,25,37–39]. In addition, laser radiation and heating may affect cells during laser based cell transfer. The impact upon landing on the acceptor surface is believed to be a key source of cell damage [17,40], and it has been shown, that the cell survival rates can be raised from 50% to 95% by increasing the thickness of hydrogel on the acceptor surface from 20 to 40 μm [12]. In addition to cushion thickness, the deceleration and shear forces exerted on the cells upon impacting the acceptor surface also depend on the velocity and size of the impacting droplets [38]. Radiation and thermal damage should be negligible for cells outside the laser focus, as near infrared radiation hardly interacts with biological material, unless energy densities are high enough for multi-photon absorption, which is only the case in the laser focus [26,30]. In addition, it has been shown, that energy absorption, and cavitation bubble expansion is much faster than energy transport through thermal conductivity, and that heating is therefore negligible outside of the laser focus [25,41].

For laser-induced transfer of aqueous liquids with femtosecond near infrared lasers pulses, it has been shown, that two consecutive liquid jets are generated at the air-liquid interface [30,31]. Approximately 1 μs after arrival of the laser pulse, a rapidly expanding, only a few micrometer thick jet appears at the interface and is ejected with velocities of 45 to 60 m/s, depending on pulse energy. At 10 to 15 μs, a second, 60 μm thick jet appears, which is expanding with velocities of only 5 to 6 m/s. While the first jet rapidly disintegrates into small fast moving micro-droplets, at small pulse energies, the second jet collapses between 30 and 40 μs. Only at laser pulse energies above approximately 5 μJ, the second jet is able to escape from the liquid and a liquid droplet with approximately 60 μm diameter is ejected towards the acceptor slide at a velocity of 3 m/s [30].

The cell-laden micro-droplets shown in Fig 2 were transferred at a laser pulse energy of 5 μJ (after the objective), which is close to the threshold energy, necessary for the second jet to escape from the liquid reservoir, reported in the literature [30]. We therefore assume that the observed 80 μm droplets are indeed the result of the first fast-moving liquid jet, while the larger 200 μm droplets result from the second jet, which ejects droplets at a 15–20 times smaller velocity [30]. If the impact at the acceptor surface is indeed the main source of cell damage, this could explain the higher survival rates observed in the larger droplets. In addition to the reduced deceleration forces because of lower droplet velocities, the larger droplets can also cushion the impact more effectively than smaller droplets. Furthermore, acceleration and shear forces during jet generation and expansion would be reduced for the wider and more slowly moving second jet. Note that the cells create heterogeneities in the liquid reservoir, which could explain, why we observe both small and large transferred micro-droplets, when operating near the threshold energy required for escape of the second jet from the hydrogel surface.

**Fig 3:**
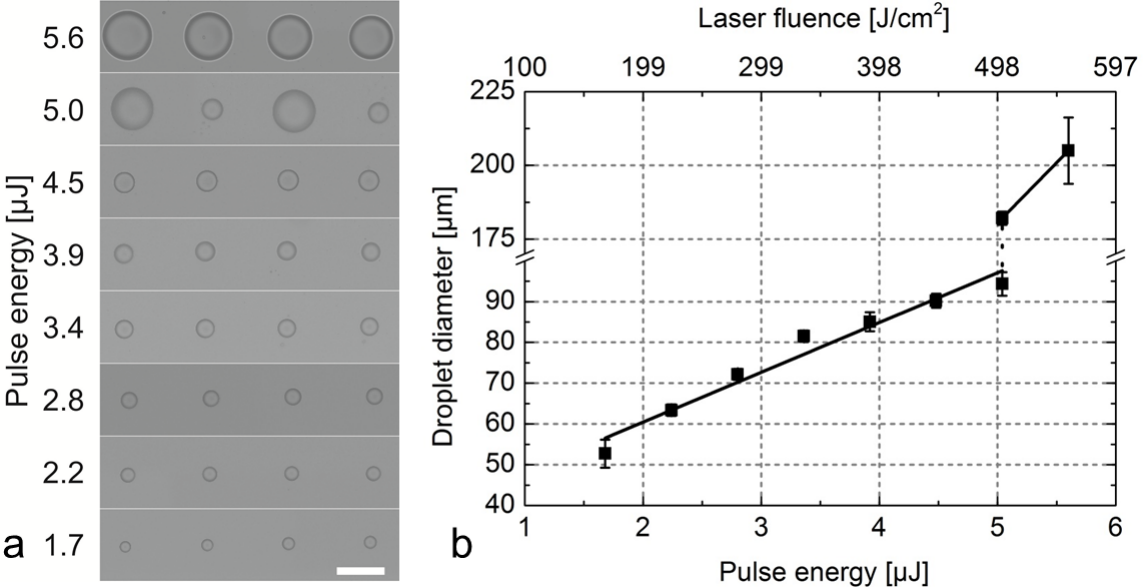
Pulse energy optimization. (a) Phase contrast microscopy images of droplet arrays printed by femtosecond laser-induced transfer on an acceptor slide with varying laser pulse energies. Scale bar = 200 μm. (b) Plot of transferred droplet diameter versus the laser pulse energy. Pulse energies were determined behind the focusing objective, which has a transmittance of 65% at 1030 nm.

To test this hypothesis, we determined the threshold energy for the transfer of large droplets in our setup using cell-free, pure hydrogel. Fig 3 shows the results of a series of experiments with increasing laser pulse energies. Up to 4.5 μJ, only small hydrogel droplets are generated, with a linear increase in droplet diameter with increasing laser pulse energy from 53 μm at 1.7 μJ to 94 μm at 5.0 μJ. At 5.0 μJ, both types of droplets are observed, and at a laser pulse energy of 5.6 μJ, only large droplets with a diameter of 205 μm appear on the acceptor surface. The results summarized in Fig 3 confirm, that the 5.0 μJ used for the cell transfer in Fig 2 are exactly at the threshold pulse energy for the escape of the second jet from the hydrogel.

**Fig 4:**
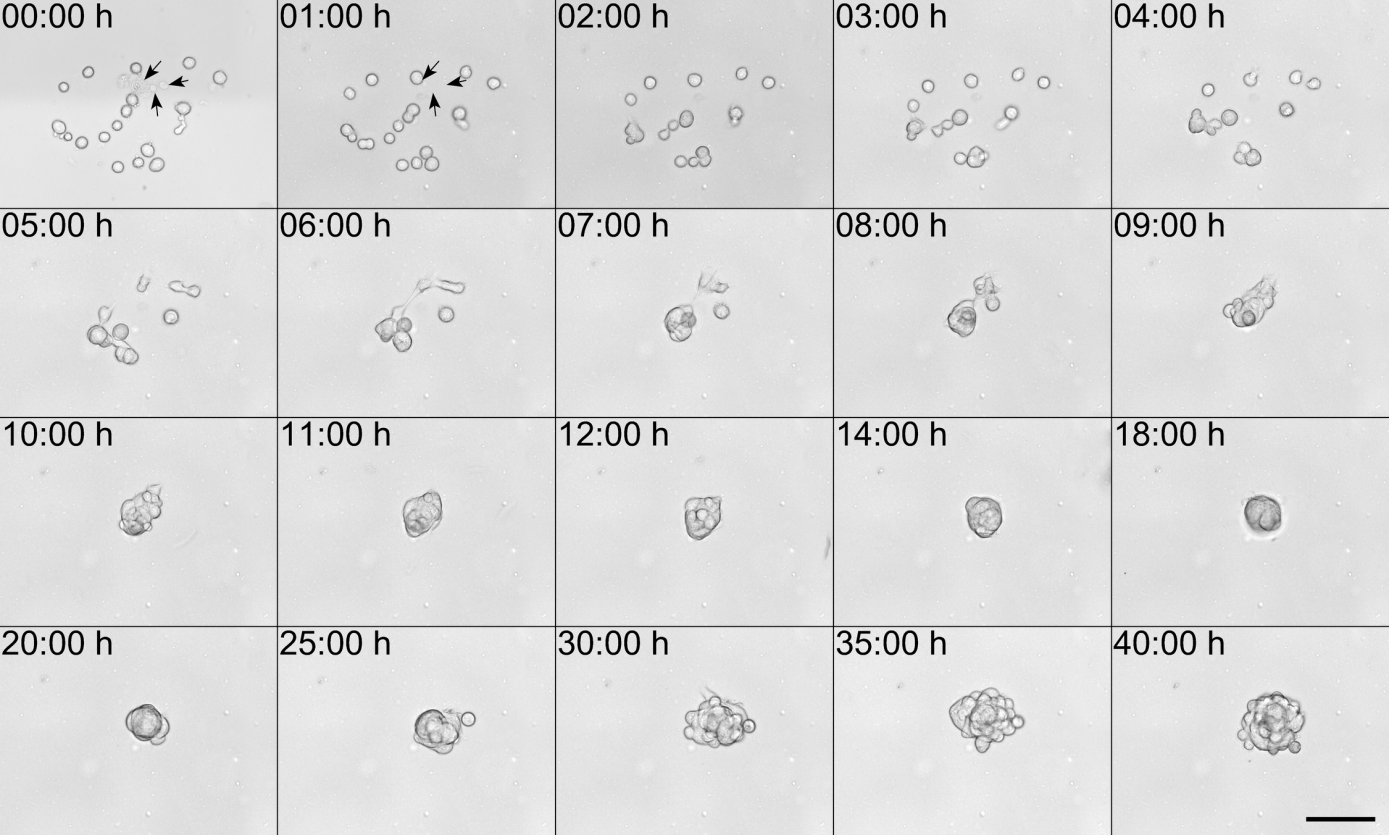
Time-lapse microscopy of cell migration and proliferation. Live cells are left to adhere to the Matrigel substrate for 15 min, whereas dead cells are washed away when adding 3 ml DMEM medium, which was gently pipetted into the dish (arrows at 0:00 and 1:00 h). The cells were monitored for a period of 40 h. After two hours of incubation, individual cells start migration and cluster formation, after 09:00 h a common cluster comprising of all cells was formed. Increase of cell number and cluster volume after 30 h indicates proliferation with a cell doubling time of 15 ± 2 hours. Scale bar corresponds to 100 μm.

Fig 4 shows mouse melanoma cells (B16F1 cell line) which were transferred in one single droplet from a histopaque reservoir supplemented with 0.2% alginate at 5.4 μJ laser pulse energy, which is above the threshold energy for the second jet. To investigate the long-term effects of laser-induced transfer on cell viability, we conducted time laps microscopy of the transferred cells over 40 hours (cf. S1 Video). Fig 4 shows the transferred cells at 15 different time-points. Immediately after transfer (00:00 h), the petri dish, which served as acceptor surface was transferred into the incubation chamber of the fluorescence microscope, and a first image was recorded (cf. materials and methods for details). To allow the melanoma cells to adhere to the acceptor substrate, the surface was coated with a 100 μm layer of Matrigel, instead of gelatin, and the cells were allowed to rest on the Matrigel coated substrate for 15 minutes, before cell culture medium was added. Note that unlike the bone marrow derived stem cells shown in Fig 2, which adhere well on pure gelatin gel, a hydrolyzed form of collagen, the most abundant protein in bone and cartilage, the melanoma cells prefer Matrigel as a substrate, because it resembles the protein composition of the basal lamina [42]. After one hour (01:00 h), the distribution and appearance of cells still resembled the appearance immediately after transfer, except for three cells which are missing and which were most likely washed off the substrate when adding the cell culture medium (indicated by arrows). These were presumably dead cells, which were not able to adhere to the Matrigel substrate. With a total cell number of 20 cells, this corresponds to a survival rate of transferred cells of 85%, for the spot shown. After two hours of incubation, the cells started to migrate towards each other and form first cell clusters, and after nine hours, all cells from the transferred droplet were forming a single cluster in the center of the image. Migration towards each other and clustering is a typical phenomenon, which is frequently observed for melanoma and other cancer cells [43]. At 30 hours, a significant increase in the cluster volume and cell number could be observed, and at 40 hours, the size of the cluster increased again, showing that the cells are able to proliferate normally, after laser-induced cell transfer. The cell proliferation rate was estimated by the increase of cluster volume after cell cluster formation was completed, 9 hours after transfer [44]. Time-lapse video microscopy revealed a cell doubling time of 15 ± 2 hours (mean value and standard deviation calculated from three cell-laden droplets) compared to a previously reported doubling time of 22 hours under standard cell culture conditions [43].

In addition to cell survival and proliferation, we checked for DNA damage using a double strand break staining kit (STA321, Cell Biolabs Inc, USA), which is based on the phosphorylation of histone H2AX. No DNA double stand breaks could be detected (data not shown). A previous bioprinting study using a 193 nm UV-laser compared the survival rates and DNA damage of a cell-laden alginate bioink with and without an additional UV absorbing gelatin layer. Post-transfer cell survival rate of 77% and 68%, respectively, and DNA damage of 10% and 21%, respectively, were reported [17]. In comparison, with 91 ± 2% survival rate and no detectable DNA double stand breaks, our film-free near-infrared fs laser printing method displays significantly higher survival rates and no DNA damage.

## Conclusions

Laser-induced transfer presents a promising approach for the fast printing of biomolecules and mammalian cells with high spatial resolution. The technique has been successfully applied to a variety of cell types, including MG63 human osteosarcoma cells, P16 pluripotent embryonal carcinoma cells, skin cell lines (fibroblasts/keratinocytes) and hMSC human mesenchymal stem cells, EA.hy926 human endothelial cells, B35 neuroblasts, and others [3,9,11–13,45,46]. The transferred cells have a high survival rate and maintain their ability to proliferation and differentiation. Nevertheless, for absorption of the laser energy, most setups rely on inorganic sacrificial absorbing thin films, and inorganic material is transferred to the printed product along with the cells. In some cases, protein based hydrogels, such as Matrigel or collagen have been used for energy absorption. However, these protein based absorbing layers require UV laser sources with wavelengths around 200 nm for effective energy absorption, which brings along the risk of DNA damage and carcinogenesis. For this reason, we have presented a new approach, which relies on non-linear absorption in the focus of femtosecond laser pulses in aqueous solution, where plasma with high electron density is generated and optical breakdown leads to efficient energy absorption. To minimize the deposition of laser energy outside of the laser focus, we have chosen a laser wavelength of 1030 nm, which uses the near infrared window, where the interaction of light with biological material is minimal. In addition, due to the fast energy deposition and expansion of the cavitation bubble, laser pulse energy is effectively converted into kinetic energy, and heating effects outside of the laser focus can be neglected. We have shown that the transferred cells have survival rates around 90%, they maintain their ability to migrate and proliferate, and show normal, cell type specific behavior after transfer. In addition, no DNA double strand breaks could be detected.

The industrial femtosecond laser used in our study is commercially available with pulse repetition rates of typically 1 MHz [47]. Assuming a number of 10 transferred cells per pulse and given that the positioning system is fast enough, a cell transfer rate of a few 10^7^ cells per second seems feasible in the future. Therefore, the used laser type, together with the presented application pave the road for a fast transfer of human cells in future bioprinting and tissue engineering applications.

## Supporting information

S1 Video Time-lapse video. Time-lapse series of B16F1 cells after femtosecond laser-induced transfer.
(AVI)
